# Supplementary material for: Distinct African Swine Fever Virus Shedding in Wild Boar Infected with Virulent and Attenuated Isolates
Source: Vaccines (Basel). 2020 Dec 16;8(4):767. doi: 10.3390/vaccines8040767 (PMC7765575; doi:10.3390/vaccines8040767)
Supplement: Supplementary file 1 [file vaccines-08-00767-s001.pdf]

Supplementary Material

Table S1. ASFV DNA detection in tissues determined by real-time PCR in the inoculated and in-contact wild boar infected with the Lv17/WB/Rie1 ASFV isolate in the attenuated group or with Arm07 ASFV isolate in the virulent group.

Tissue	Attenuated group		Virulent group	
	**Orally inoculated** Ct values of real-time PCR (± SD) [1]	**In-contact** Ct values of real-time PCR (± SD) [1]	**Intramuscularly inoculated** Ct values of real-time PCR (± SD) [1]	**In-contact** Ct values of real-time PCR (± SD) [1]
Heart	37 ± 5	37 ± 2	25 ± 7	23 ± 3
Lung	37 ± 5	Neg	22 ± 3	22 ± 2
Kidney	38 ± 5	Neg	24 ± 5	22 ± 2
Liver	38 ± 4	Neg	23 ± 7	20 ± 2
Urinary bladder	37 ± 5	Neg	26 ± 6	26 ± 3
Spleen	36 ± 6	39 ± 2	22 ± 6	20 ± 4
Lymph nodes[2]	37 ± 1	39 ± 1	25 ± 5	23 ± 2
Tonsil	no data	no data	25 ± 8	22 ± 7
Bone Marrow	38 ± 6	Neg	24 ± 8	22 ± 4
Brain	38 ± 5	Neg	25 ± 7	23 ± 2

[1]Mean cycle threshold (± standard deviation) in quantitative PCR of the indicated tissue. [2]Lymph nodes: These CT values were obtained from a pool of eight different lymph nodes: renal, mediastinal, retropharyngeal, mesenteric, preescapular, gastrohepatic, inguinal and mandibular lymph nodes.

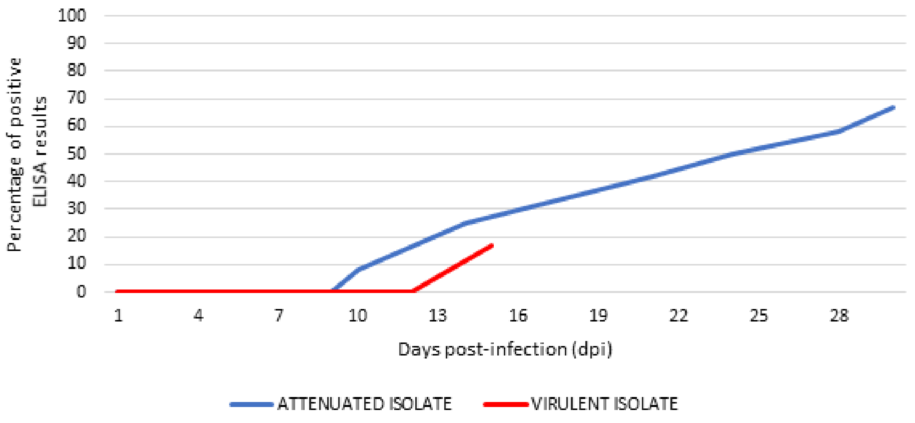

Figure S1. Percentage of positive animals for antibody detection in serum samples determined by commercial ELISA kit for the detection of p72 ASFV antigen (Ingenasa-Ingenzim PPA Compac K3; Ingenasa, Madrid, Spain) in the attenuated group (blue line) and in the virulent group (red line).
